# Population Genomics Provides Insights Into Genomic Features of Inbreeding Depression in *Arma Chinensis*

**DOI:** 10.1111/eva.70107

**Published:** 2025-06-08

**Authors:** Bin Li, Kangkang Song, Zixian Wu, Xiaohua Zhang, Haozhen Li, Long Yang

**Affiliations:** ^1^ Agricultural Big‐Data Research Center and College of Plant Protection Shandong Agricultural University Tai'an China; ^2^ The Key Laboratory of Plant Development and Environmental Adaptation Biology, Ministry of Education, School of Life Sciences Shandong University Qingdao China; ^3^ Ganzhou Company of Jiangxi Tobacco Corporation Ganzhou Jiangxi Province China

**Keywords:** *Arma chinensis*, genetic diversity, inbreeding depression, phylogenomic analysis, runs of homozygosity

## Abstract

*Arma chinensis*, a predatory insect renowned for its prey diversity in East Asia, is effective in controlling agricultural and forestry pests. However, after introducing field populations into indoor subcultures, features of inbreeding depression have surfaced within these populations. Clarifying the molecular genetic mechanism of inbreeding depression of *A. chinensis* is of great significance for its population protection. In this study, phylogenomic analysis revealed that the genus *Arma* shared a common ancestor with *Halyomorpha* and *Nezara* in the Pentatomidae family around 63.62 million years ago. Based on whole‐genome resequencing of three consecutive inbred generations of *A. chinensis* , we investigated the genomic features of inbreeding depression. We observed an accumulation of long runs of homozygosity and extreme variations in nucleotide diversity across generations, collectively affecting 111 genes and multiple biological processes, such as sequence‐specific DNA binding, synapse organization, and transcription regulatory region binding. These genomic changes suggest that successive inbreeding may disrupt normal physiological functions, potentially impairing gene expression, neural signaling, and sensory organ development. In conclusion, our study clarifies the evolutionary position of *A. chinensis* , highlights the genetic consequences of inbreeding, and emphasizes the importance of preserving genetic diversity in natural populations for long‐term survival and adaptability.

## 1 Introduction

**FIGURE 1:**
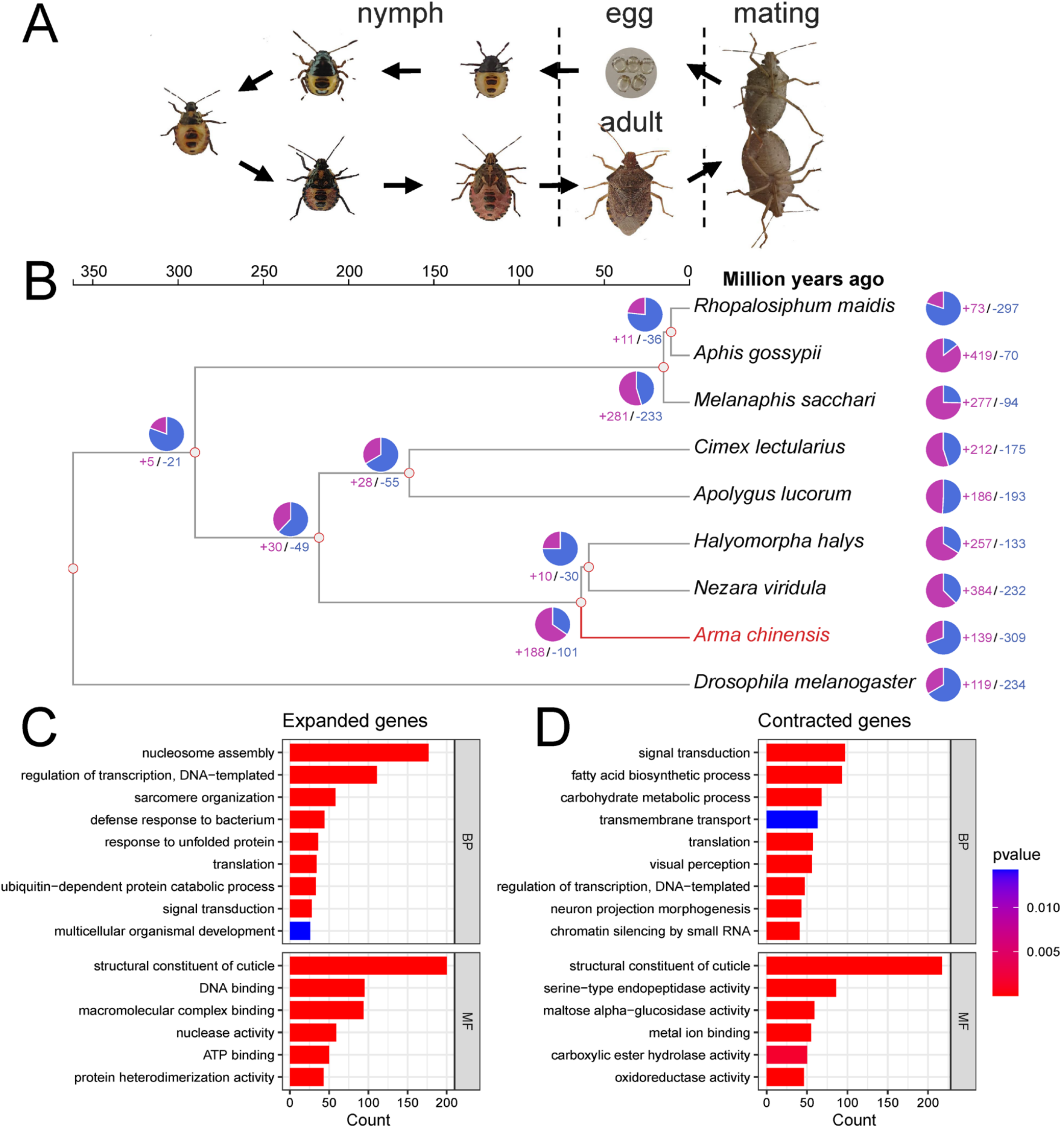
Life cycle and comparative genomics analysis of *A. chinensis* . (A) The life cycle starts from eggs, progresses through five nymphal stages, and develops into adulthood. Mating adults produce eggs, thus completing the cycle. (B) Phylogenomic relationship of 9 species ( *A. chinensis* , *A. gossypii* , *A. lucorum* , *C. lectularius* , *D. melanogaster* , *H. halys* , *M. sacchari* , *N. viridula* , *R. maidis* ). The divergence times are labeled at the top. The numbers on each branch represent expansion (purple) and contraction (blue) of gene families. GO enrichment of (C) expanded and (D) contracted gene families in *A. chinensis* .

*Arma chinensis* (order: Hemiptera; family: Pentatomidae; Figure 1A) is a predatory insect renowned for its prey diversity, effectively suppressing pests from families such as Lepidoptera, Coleoptera, Hymenoptera, and Hemiptera (Deyu et al. 2012; M. Pan et al. 2019; H. Wu et al. 2019). *A. chinensis* is predominantly found in East Asia, including China, the Korean Peninsula, Japan, and Mongolia (M.‐Z. Pan et al. 2022; Rider et al. 2002), exhibiting remarkable environmental adaptability. Given suitable ecological conditions, *A. chinensis* can establish a natural population in the released area, thereby facilitating long‐term pest management (M.‐Z. Pan et al. 2022). Therefore, *A. chinensis* stands out as a beneficial predator capable of controlling pests on a broad scale, simultaneously mitigating the harms associated with chemical pest management and advancing the development of comprehensive pest control systems (Shen et al. 2018). Nevertheless, following the introduction of field populations into indoor cultures, features of inbreeding depression, including the decrease of egg hatching rate and the increase of time needed to reach adulthood, have gradually emerged within the *A. chinensis* populations (Shaoming et al. 2021; Zhu et al. 2022). This situation poses considerable obstacles to the propagation and agricultural utilization of this beneficial insect, emphasizing the urgency of exploring its genetic consequences.

Traditionally, breeding strategies often involve measures, including inbreeding, to generate homozygous or inbred lines, thereby fixing traits of interest (Burgarella et al. 2019). However, sustained inbreeding over numerous generations, particularly in small populations, could lead to several genetic consequences. These include the loss of genetic diversity (Eszterbauer et al. 2015), fixation of deleterious alleles (Assaf et al. 2015; Smeds and Ellegren 2023), increased genetic load (Marsden et al. 2016; Wang et al. 2017), and ultimately, inbreeding depression (D. Charlesworth and Willis 2009; Han et al. 2021).

Inbreeding has been found to have adverse effects on insects (Kuriwada et al. 2010; Peng et al. 2015; Woldemelak 2024). Current research on inbreeding depression of insects predominantly focuses on the living processes and biological consequences of inbreeding (Collet et al. 2020; Pilakouta et al. 2015). However, there remains a lack of exploration of the molecular genetic underpinnings of the whole genome, including the three aspects of identifying specific genes or pathways affected, understanding their modes of action, and their interactions with adaptation in insect species. Consequently, a comprehensive, genome‐wide examination of the genetic mechanisms that underlie inbreeding depression of insects is imperative for a more nuanced understanding of this phenomenon.

This study aims to investigate the genetic basis of inbreeding depression during indoor rearing of *A. chinensis* by integrating genomics with whole‐genome resequencing. First, based on the reported *A. chinensis* genome assembly (Fu et al. 2024), we conducted phylogenomic analysis to determine its evolutionary status. Subsequently, an artificial inbreeding test line was established under indoor conditions, followed by whole‐genome resequencing. This allowed us to delve into the genetic underpinnings of each generation, thereby elucidating the molecular genetic mechanisms driving inbreeding depression in *A. chinensis* . Leveraging the insights from this study, we aim to refine methods for rejuvenating the *A. chinensis* population, mitigate the negative impacts of inbreeding, and enhance its indoor breeding for optimized application in biological control.

## 2 Materials and Methods

### 2.1 Gene Family Identification, Phylogenomic Analysis, and Gene Family Expansion and Contraction Analysis

Protein sequences from *A. chinensis* , 7 other Hemiptera species ( *Aphis gossypii* , *Apolygus lucorum*, *Cimex lectularius* , *Halyomorpha halys* , *Melanaphis sacchari* , *Nezara viridula* , *Rhopalosiphum maidis* ), and 1 outgroup ( *Drosophila melanogaster* ) were analysed on the OrthoVenn3 website (Table 1) (Sun et al. 2023). The longest transcript was selected to represent each gene with multiple isoforms. Protein sequences from *A. chinensis* and 8 other species were used for family classification using OrthoFinder (Emms and Kelly 2019) with an E value of 1e‐2 and an inflation value of 1.5. Single‐copy orthologous clusters were aligned using MUSCLE (Edgar 2004), and conserved sequences were extracted and trimmed with trimAI (Capella‐Gutiérrez et al. 2009). FastTree (Price et al. 2010) was then employed to infer the phylogenomic tree among species via the maximum likelihood method (Felsenstein 1981). Gene family contraction and expansion analyses were conducted with CAFÉ5 (Mendes et al. 2021) on the OrthoVenn3 website. CAFÉ5 uses a threshold of 0.05 and comprehensively judges the significant expansion or contraction of gene families based on Family‐wide *p*‐value, Viterbi *p*‐value, and Cut *p*‐value. Estimates were based on fossil times ( *H. halys* — *N. viridula* to 59.15 Mya, *A. gossypii* — *A. lucorum* to 290.05 Mya, *C. lectularius* — *H. halys* to 217.31 Mya, *R. maidis* — *N. viridula* to 290.05 Mya, *D. melanogaster* — *R. maidis* to 361.53 Mya, *D. melanogaster* — *A. gossypii* to 361.53 Mya) obtained from TimeTree (Kumar et al. 2017) using the OrthoVenn3 website. The expanded and contracted gene families in *A. chinensis* as specific genes were constructed for GO enrichment analysis.

**TABLE 1:** List of insect protein sequences used in the comparative genomics analysis.

Species	from (default from NCBI)
*Halyomorpha halys*	GCF_000696795.2
*Nezara viridula*	GCA_928085145.1
*Rhopalosiphum maidis*	OrthoVenn3 website (https://orthovenn3.bioinfotoolkits.net/)
*Aphis gossypii*	GCF_020184175.1
*Melanaphis sacchari*	GCF_002803265.2
*Apolygus lucorum*	GCA_009739505.2
*Cimex lectularius*	GCF_000648675.2
*Drosophila melanogaster*	GCA_000001215.4
*Arma chinensis*	GCA_040285775.1

### 2.2 Samples for Whole‐Genome Resequencing

Parent samples of *A. chinensis* were collected from the field in Linyi City, Shandong Province, China (35°50 N, 118°38 E), and cultivated indoors by continuous free inbreeding between the same generation. The indoor feeding temperature was kept at 26°C ± 1°C, the relative humidity was 66% RH, and the photoperiod was 16 L: 8 D. A total of 18 adult male individuals (9 F1, 4 F2, and 5 F3) from three consecutive inbreeding generations were selected, and the viscera were removed for subsequent sequencing. These samples were used for the construction of 150‐bp paired‐end libraries. The genomes of these 18 individuals were then resequenced using the Illumina NovaSeq 6000 platform (Illumina, CA, USA), yielding 189.83 Gb of clean reads.

### 2.3 Genetic Variation Detection

Whole‐genome resequencing reads were trimmed by Trimmomatic (v0.39) (Bolger et al. 2014) and assessed by FastQC (v0.12.1). Clean reads were mapped to the *A. chinensis* genome assembly using BWA (v0.7.17‐r1188) (Li and Durbin 2009). Alignment reads were marked as duplicated, sorted, and indexed using SAMtools (v1.20) (Li 2011). Variant detection was performed for each sample using GATK (v3.8.0) with default parameters (McKenna et al. 2010). BCFtools (v1.9) (Danecek et al. 2021) was used to identify SNPs. The SNPs were filtered using VCFtools (v0.1.16) (∔mac 1—min‐alleles 2—max‐alleles 2—minGQ 30—minQ 300—maxDP 80) (Viluma et al. 2022).

### 2.4 PCA and Phylogenomic Analysis for *A. chinensis* Populations

PCA analysis was performed with the final filtered VCF files using PLINK (v1.90b6.21) (Purcell et al. 2007) and visualized by the R package scatterplot3d (v0.3–44) (Ligges and Maechler 2003). IQ‐TREE (v2.2.6) (Nguyen et al. 2015) was used to construct the phylogenomic tree. ModelFinder (Kalyaanamoorthy et al. 2017) was used for model selection, and the best model was obtained as mtZOA+F + G4, and then the maximum likelihood method was used to construct the evolutionary tree using this best model. The phylogenomic tree was visualized by the R package ggtree (v3.11.2) (Xu et al. 2022).

### 2.5 Runs of Homozygosity and Nucleotide Diversity Detection

Following the recommendation by Meyermans et al. (2020), linkage disequilibrium (LD) and minor allele frequency (MAF) trimming were not conducted prior to detecting runs of homozygosity (ROH) (Meyermans et al. 2020). ROH analysis was performed by PLINK (∔homozyg‐window‐snp 5—homozyg‐kb 5). F_ROH_ is defined as the proportion of the autosomal genome in ROHs exceeding a specified length (McQuillan et al. 2008). ROH‐related features, including the distribution of F_ROH_, number of ROHs, and average length of ROHs, were visualized by R package ggplot2 (v3.5.1) (Ginestet 2011). The genes covered by ROH regions exceeding 400 kb specific to the F3 generation of *A. chinensis* as specific genes were executed for GO enrichment analysis. Nucleotide diversity for SNPs was detected using VCFtools (∔site‐pi). The independent samples t‐tests were performed using the t_test function from the rstatix package (v0.7.2) (Kassambara 2023). Manhattan plots were generated with the ggplot2 package, based on a random sample of 1% of SNPs from each generation. The sankey plot was visualized using the sankeyNetwork function from the networkD3 package (v0.4) (Gandrud et al. 2017), illustrating the transitions of SNPs between different nucleotide diversity bins across the three generations. GO functional enrichment analysis was performed on genes covered by highly conserved/diverse sites (π = 0/1) specific to F2 and F3 populations.

### 2.6 GO Functional Enrichment Analysis

Multiple GO analyses were conducted to explore the specific impacts of various genomic changes resulting from inbreeding. Multiple GO analyses were constructed with the purpose of exploring the specific impacts caused by multiple types of genomic changes and their potential consequences. The organism database for *A. chinensis* was generated by the R package AnnotationForge (v1.45.0) (Carlson et al. 2016). GO functional enrichment analysis of specific genes was performed by the R package ClusterProfiler (v4.11.1) with default parameters (T. Wu et al. 2021; Yu et al. 2012). The specific genes have been defined in the preceding Materials and Methods. The enrichment results were visualized by the R package ggplot2.

## 3 Results

### 3.1 Evolution History and Comparative Analysis of *A. chinensis*

Based on the chromosome‐level genome assembly of *A. chinensis* reported by Fu et al. (Fu et al. 2024), we further determined its evolutionary position. We performed phylogenomic analysis using protein sequences from *A. chinensis* , 7 other Hemiptera species, and 1 outgroup ( *D. melanogaster* ) (Table 1). All protein‐coding genes were clustered into 17,057 orthogroups based on sequence homology. Additionally, we identified a total of 4772 gene families shared among all 9 species, along with 402 species‐specific gene families in *A. chinensis* (Figure S1). GO enrichment of these genes indicated the enrichment of myofibril assembly, DNA integration, DNA binding, multicellular organismal development, and structural constituent of cuticle (Figure S1).

The phylogenomic tree was constructed using 1194 single‐copy orthologs (Figure S2). Phylogenomic analysis revealed that the genus *Arma* shared a common ancestor with *Halyomorpha* and *Nezara* within the Pentatomidae family around 63.62 million years ago (Figure 1B). The dynamics of gene family detected 139 and 309 gene family expansions and contractions in the *A. chinensis* genome, respectively. GO enrichment analysis indicated that expanded gene families in *A. chinensis* were associated with structural constituent of cuticle, nucleosome assembly, regulation of transcription and DNA‐templated, DNA binding, and macromolecular complex binding (Figure 1C). Additionally, the analysis indicated that contracted gene families were related to structural constituent of cuticle, signal transduction, fatty acid biosynthetic process, serine‐type endopeptidase activity, and carbohydrate metabolic process (Figure 1D).

### 3.2 Inbreeding Depression Accompanied by Accumulation of Long Runs of Homozygosity Regions

To investigate the molecular mechanisms driving inbreeding depression in *A. chinensis* , whole‐genome resequencing was performed on 18 adults from three consecutive inbreeding generations (9 F1, 4 F2, and 5 F3) reared indoors. The above process yielded 189.83 Gb of high‐quality data (~10.87× coverage per sample), resulting in the identification of 3,158,715 qualified SNPs upon alignment with the *A. chinensis* reference genome. Both phylogenomic and PCA analyses consistently showed a weak clustering pattern, revealing small population genetic shifts associated with inbreeding (Figures S3–S4).

**FIGURE 2:**
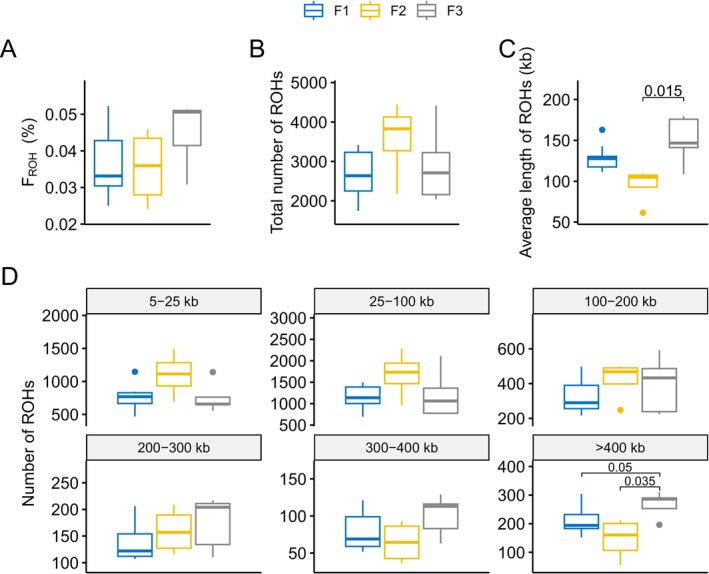
Statistics of ROH features across three successive inbred generations of *A. chinensis* . (A) F_ROH_, (B) total number of ROHs, (C) average length of ROHs, and (D) number of ROH regions for different fragment lengths in three generations. *p*‐values from independent samples t‐tests are indicated where *p* < 0.05.

Subsequently, ROH features of three successive inbreeding generations of *A. chinensis* were detected. An increasing trend in F_ROH_ was observed, indicating the emergence of inbreeding depression (Figure 2A). While the total number of ROH regions did not show an obvious trend (Figure 2B), the average length of ROHs significantly increased in the F3 generation (Figure 2C), leading to an accumulation of total ROH length and a subsequent rise in F_ROH_. This accumulation was primarily driven by a significant increase in long ROH regions (> 400 kb) (Figure 2D), which covered 2229 genes specific to the F3 generation.

To assess the impact of inbreeding on these long ROH regions, we performed GO enrichment analysis on genes covered by ROHs exceeding 400 kb in the F3 generation (Figure S5). The analysis revealed significant enrichments in critical biological processes, including hydrolase activity, cellular response to nitrogen compounds, immune system development, sensory perception, immune effector processes, negative regulation of cell differentiation, and male gamete generation. These findings suggest widespread genomic and phenotypic effects associated with the accumulation of long ROH regions due to inbreeding.

### 3.3 Extreme Variations in Nucleotide Diversity (*π*) Caused by Inbreeding

**FIGURE 3:**
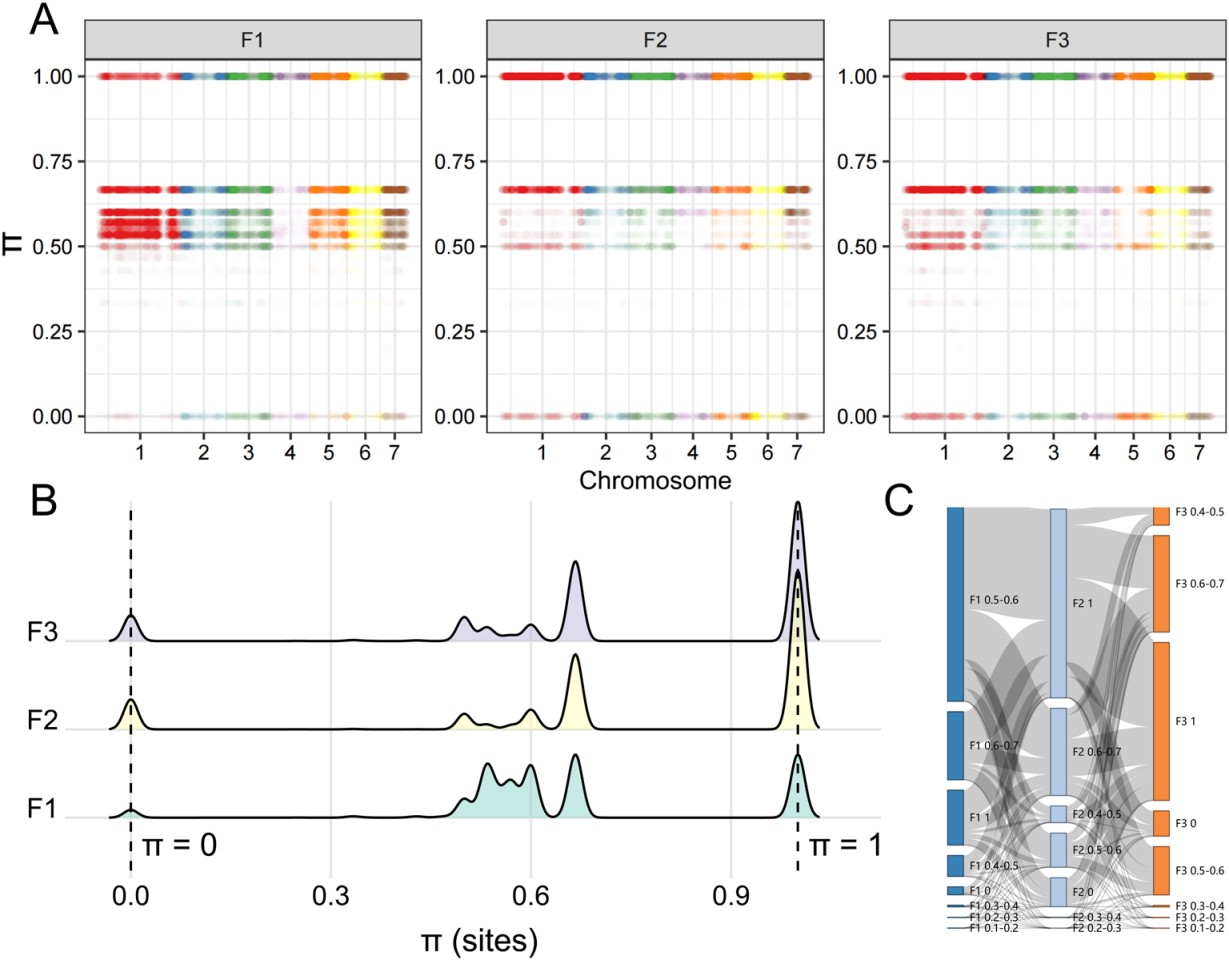
Nucleotide diversity in three successive inbred generations of *A. chinensis* . (A) Manhattan plots and (B) kernel density estimates show nucleotide diversity distribution patterns. In the Manhattan plot, different colors represent different chromosomes, with each dot corresponding to a SNP locus. (C) Sankey plot shows trends in nucleotide diversity changes across the three generations.

After undergoing three consecutive generations of inbreeding, distinct nucleotide diversity patterns have emerged (Figure 3A,B). The F1 generation exhibits a relatively dispersed nucleotide diversity distribution, with a higher proportion falling between 0.25 and 0.75. In contrast, the F2 and F3 generations exhibit clear peaks at two extremes: π = 0 for highly conserved sites and *π* = 1 for highly diverse sites. Specifically, extreme sites (*π* = 0 and *π* = 1) in the F2 generation largely inherit from non‐extreme sites in the F1 generation, and these SNP variations become fixed in the F3 generation (Figure 3C). On one hand, the emergence of highly diverse sites suggests potential for novel genetic combinations and adaptations. On the other hand, the appearance of highly conserved sites implies a loss of diversity, likely due to the fixation of specific alleles and a subsequent narrowing of the genetic pool.

To further elucidate these findings, GO enrichment analyses were performed for genes covered by these sites. The highly conserved sites specific to F2 and F3 generations were enriched in processes related to chemotaxis, regulation of anatomical structure size, hormone levels, developmental growth, transcription factor binding, secretion, neuron projection development, and postembryonic morphogenesis (Figure S6). Conversely, the highly diverse sites showed enrichment in processes involving cell fate commitment, anion transport, synaptic signaling, DNA‐binding transcription factor activity, sensory organ morphogenesis, and chemical synaptic transmission (Figure S7). This suggests that while breeding reinforces certain genetic traits, it may also trigger compensatory mechanisms potentially to mitigate the negative effects of inbred depression (Zhang et al. 2023).

In conclusion, our observations highlight the dual nature of the inbreeding process. While it can fix parental genetic variations, it simultaneously reduces overall genetic diversity by strengthening conserved genetic traits. The GO enrichment analysis provides insights into the specific biological processes affected by these genetic changes, shedding light on the complex interplay between genetic variation and conservation in the context of inbred breeding.

### 3.4 Multiple Genomic Consequences Induced by Consecutive Inbreeding

**FIGURE 4:**
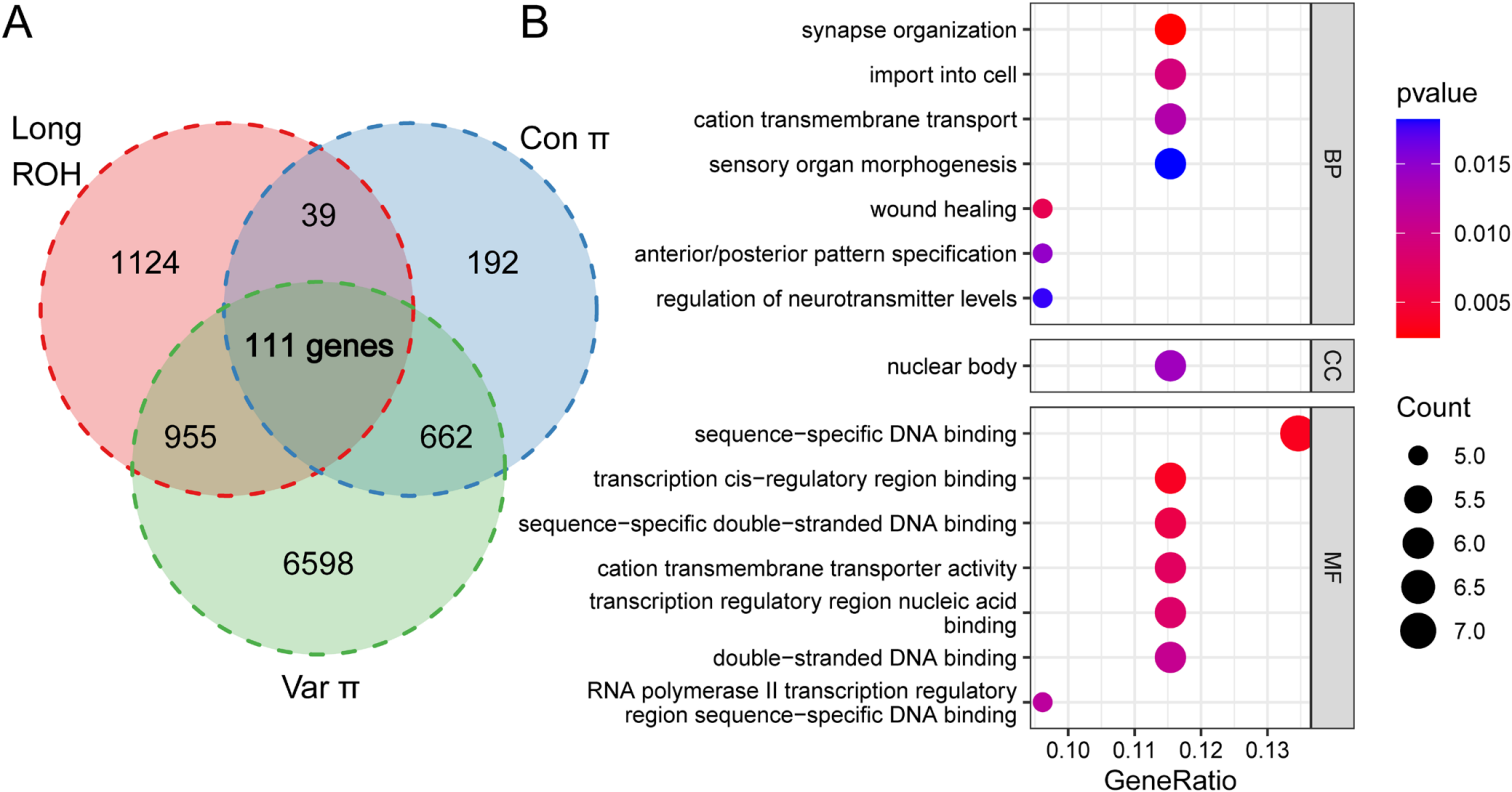
Overlapping genes from multianalyses and GO enrichment. (A) Genes identified through multiple analyses: Conserved π sites specific to F2 and F3 generations, variant π sites specific to F2 and F3 generations, and long ROH regions (> 400 kb) specific to the F3 generation. (B) GO enrichment for these overlapping genes.

Inbreeding has led to multiple genomic consequences, including the accumulation of Long ROH regions and the emergence of extreme nucleotide diversity sites. These processes have collectively impacted 111 genes (Figure 4A). GO enrichment analysis reveals that these genes are significantly enriched in functions related to sequence‐specific DNA binding, synapse organization, transcription regulation, cation transmembrane transport, nuclear body processes, sensory organ morphogenesis, wound healing, and neurotransmitter regulation (Figure 4B), highlighting their crucial roles in diverse biological processes and molecular functions potentially disrupted by the detrimental effects of successive inbreeding.

## 4 Discussion

*Arma chinensis*, a predatory natural enemy insect with a broad prey range, has effective control over agricultural and forestry pests. Fu et al. (2024) reported a high‐quality, chromosome‐level genome assembly for *A. chinensis* , spanning 986 Mb and containing 20,853 predicted protein‐encoding genes (Fu et al. 2024). Based on this, we further investigated the genome evolution and divergence of *A. chinensis* . Using 1194 single‐copy orthologs from 9 insect species, phylogenomic analysis revealed that the genus *Arma* shared a common ancestor with *Halyomorpha* and *Nezara* within the Pentatomidae family around 63.62 million years ago (Figure 1B). Overall, the *A. chinensis* genome provides a valuable resource for downstream analyses.

Previous research on inbreeding has predominantly centered around the living processes and biological consequences of inbreeding. For example, inbreeding of monarch butterflies led to higher mortality and shorter lifespan of offspring Mongue et al. (2016). Here we established inbreeding lines of *A. chinensis* over three successive generations to investigate the precise consequences of inbreeding on the genome of an insect. Our results indicate that inbreeding exerts a profound influence on the entire genome of *A. chinensis* , interfering with various biological processes and cellular functions that are vital for development, differentiation, and growth.

As inbreeding generations progressed, the increasing trend of F_ROH_ indicated the growth of homozygous segments due to inbreeding (McQuillan et al. 2008). Specifically, the steady increase in F_ROH_ over the generations was attributed to the significant increase in the average length of ROHs in the F3 generation (Figure 2C), which was driven primarily by the significant increase in long ROH regions (> 400 kb) (Figure 2D). Furthermore, there was a notable increase in both the frequency of highly conserved sites (*π* = 0) and highly diverse sites (*π* = 1) throughout the genome, which was particularly evident in the F2 and F3 populations. This transformation indicates substantial alterations in nucleotide diversity, reflecting the profound genetic repercussions of continued inbreeding (Ørsted et al. 2019; Zhang et al. 2023).

It is worth noting that the rapid emergence and stabilization of numerous highly conserved sites within a short timeframe suggests a potential loss of genetic diversity, which could pave the way for a subsequent genetic bottleneck effect (Ali and Roossinck 2008; Roy 2022). The emergence of a population bottleneck would further exacerbate the loss of genetic diversity through intensified genetic drift (B. Charlesworth 2009; Grossen et al. 2020; Lucena‐Perez et al. 2021). These observed genetic shifts underscore the fragility of genetic diversity, particularly within genetically isolated subpopulations (Exposito‐Alonso et al. 2022). This fragility underscores an urgent need for external genetic inflow as a mitigating measure against the potential effects of prolonged inbreeding (Garcia‐Cisneros et al. 2016; Stevens et al. 2018). The quality control, which included transferring newly molted adults to the next‐generation adult cage, could help recover an inbred colony of *Bagrada hilaris* (Ivey et al. 2023). Consequently, it is advisable to introduce external populations during insect rearing in laboratory cultures to alleviate problems caused by inbreeding depression (Hedrick and Garcia‐Dorado 2016; Johnson et al. 2010). Specifically, laboratories should periodically introduce new individuals from wild populations into their cultures to increase genetic diversity, regularly monitor genetic diversity using genomic tools like whole‐genome resequencing, and maintain larger population sizes to mitigate the negative effects of inbreeding.

The emergence of highly diverse sites suggests potential for new genetic combinations and adaptations. While breeding reinforces certain genetic traits, it may also trigger compensatory mechanisms potentially to mitigate the negative effects of inbreeding depression. However, this could disrupt original field‐adaptive genetic combinations. Additionally, the accumulation of long ROH regions (> 400 kb) coupled with reduced genetic diversity due to inbreeding may further compromise the population's resilience and adaptability to environmental perturbations. These processes collectively affect 111 genes in the *A. chinensis* genome. GO enrichment analysis reveals significant associations with various biological processes and functions, including sequence‐specific DNA binding, synapse organization, transcription regulation, cation transmembrane transport, nuclear body processes, sensory organ morphogenesis, wound healing, neurotransmitter regulation, among others. In a recent study of mosquitoes, many genomic variations were determined by genome resequencing, which affected the functions of mosquito digestion, development, and innate immunity (Acharya et al. 2024). These findings underscore the potential for widespread biological dysfunction resulting from inbreeding (Zhang et al. 2023).

## 5 Conclusions

In summary, we determined the evolutionary position of *A. chinensis* based on its chromosome‐level genome assembly (Fu et al. 2024). Whole‐genome resequencing of three consecutive inbred generations of *A. chinensis* revealed profound genetic consequences of inbreeding. These findings highlight the importance of preserving genetic diversity, particularly in genetically isolated subpopulations. These insights can inform conservation efforts to rejuvenate *A. chinensis* populations, mitigate the negative impacts of inbreeding, and refine indoor breeding practices for improved biological control applications.

## Conflicts of Interest

The authors declare no conflicts of interest.

## Supporting information


FIGURES S1–S7

## Data Availability

The raw data of whole genome resequencing were deposited in the NCBI Sequence Read Archive, with BioProject accession numbers PRJNA1111265.
