# Supplementary material for: Population Genomics Provides Insights Into Genomic Features of Inbreeding Depression in Arma Chinensis
Source: Evol Appl. 2025 Jun 8;18(6):e70107. doi: 10.1111/eva.70107 (PMC12146214; doi:10.1111/eva.70107)
Supplement: Supplementary file 1 — FIGURES S1–S7 [file EVA-18-e70107-s001.zip › Supplementary Figures.docx]

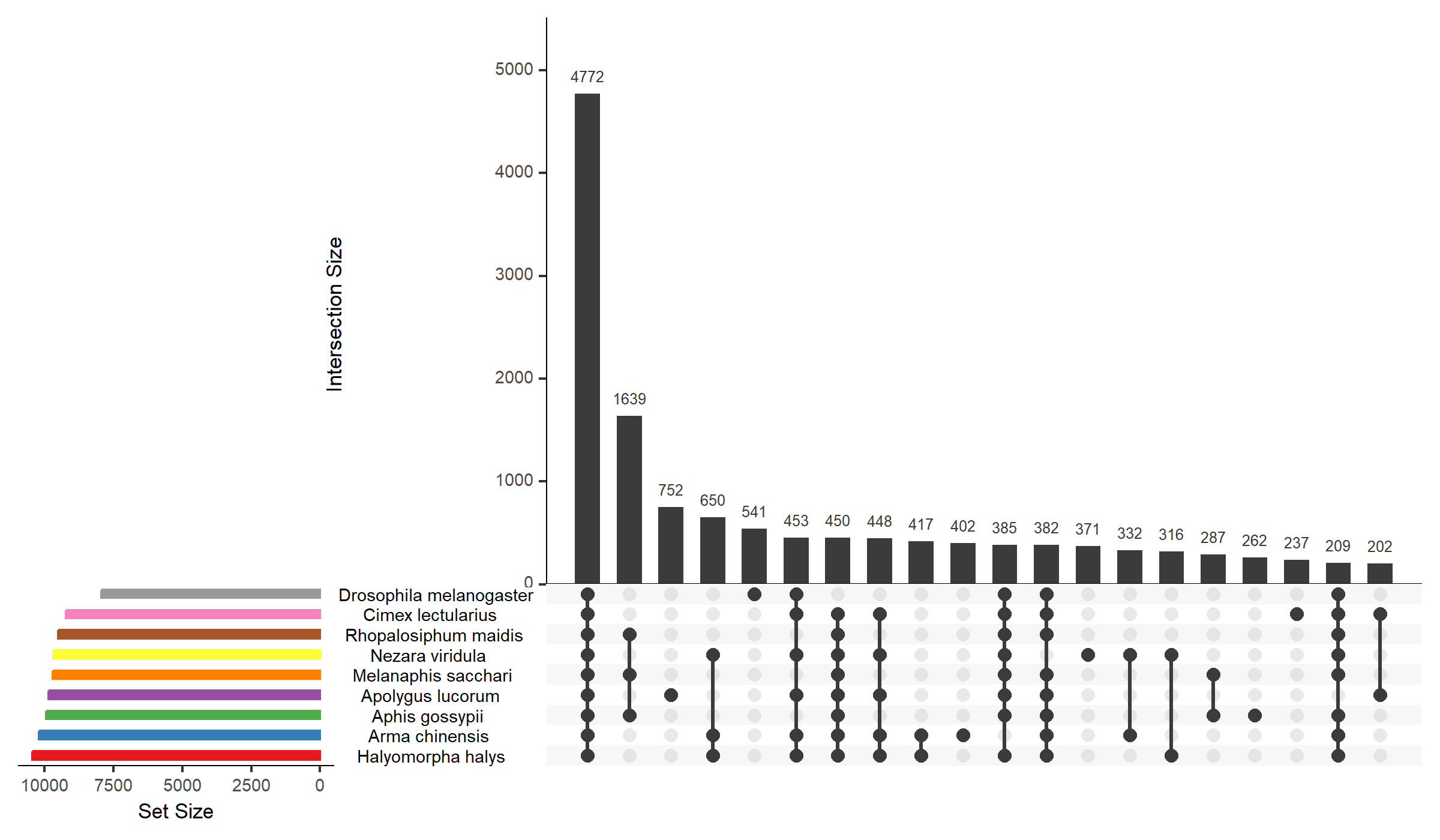


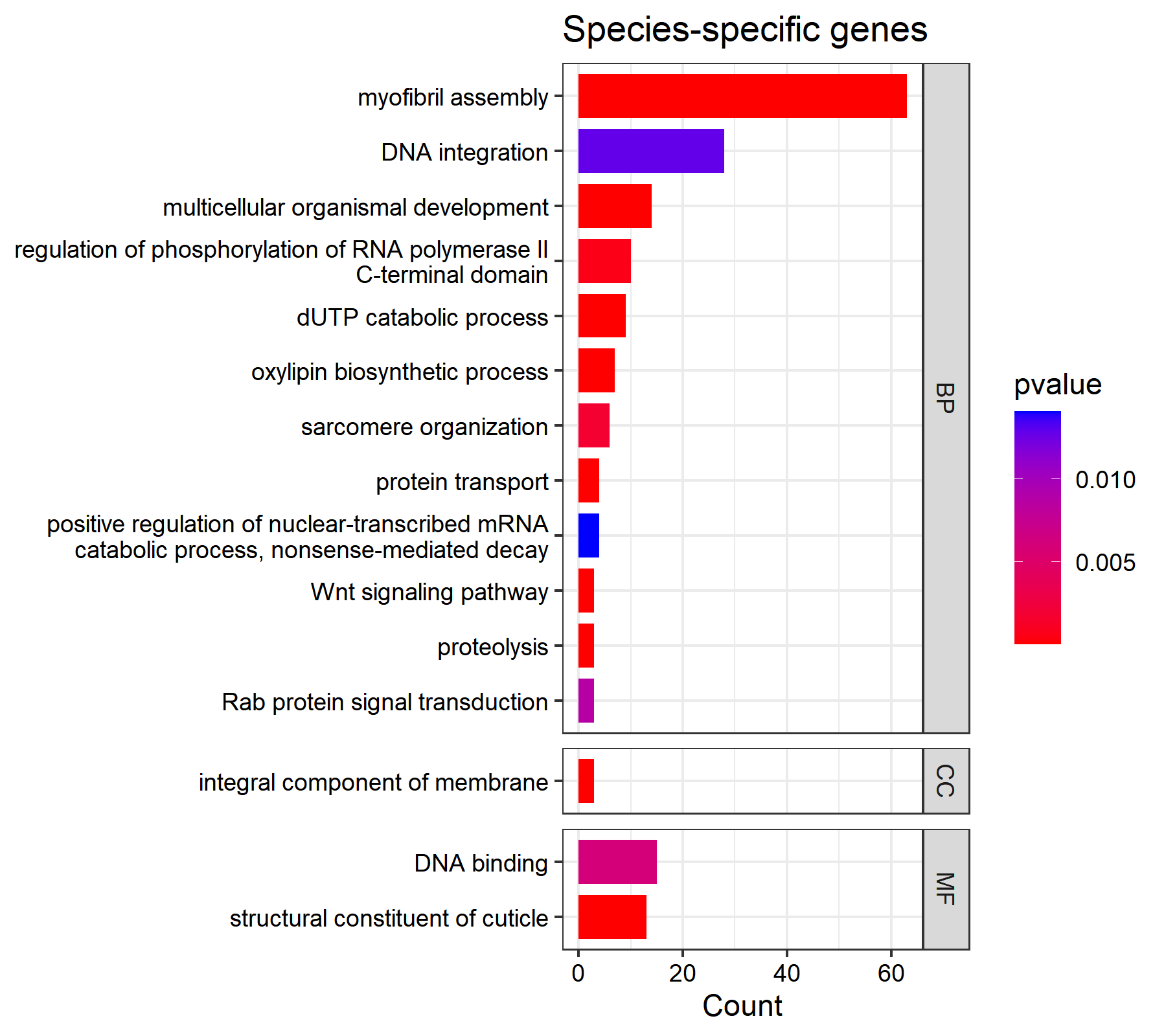


### Figure S1 Upset diagram of orthogroups among 9 species (up). GO enrichment of species-specific genes in *A. chinensis* (below).


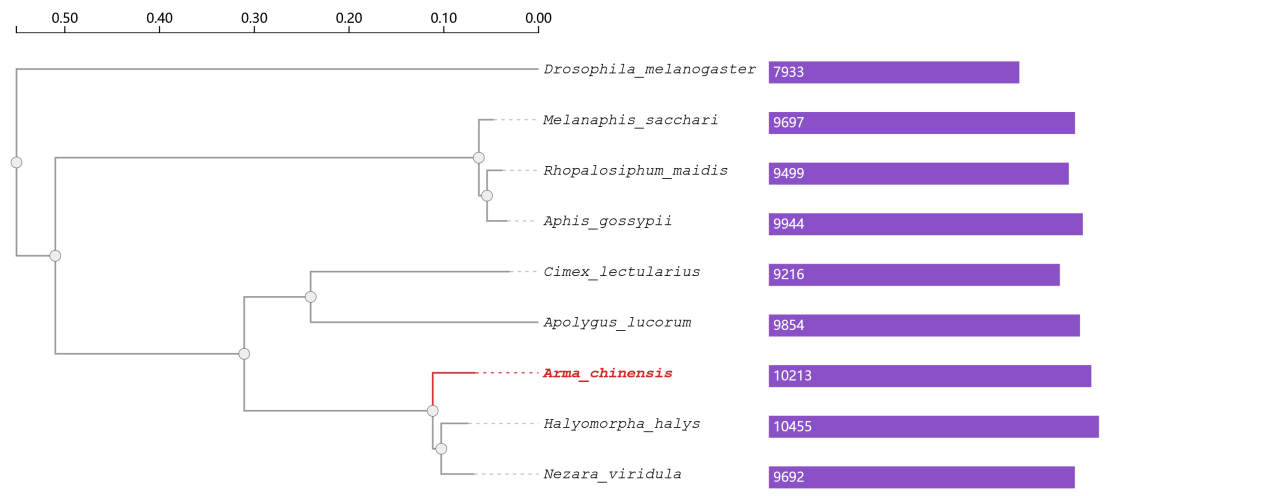


### Figure S2 Phylogenetic relationship of 9 species, with the number of orthogroups labeled on the right-hand side.


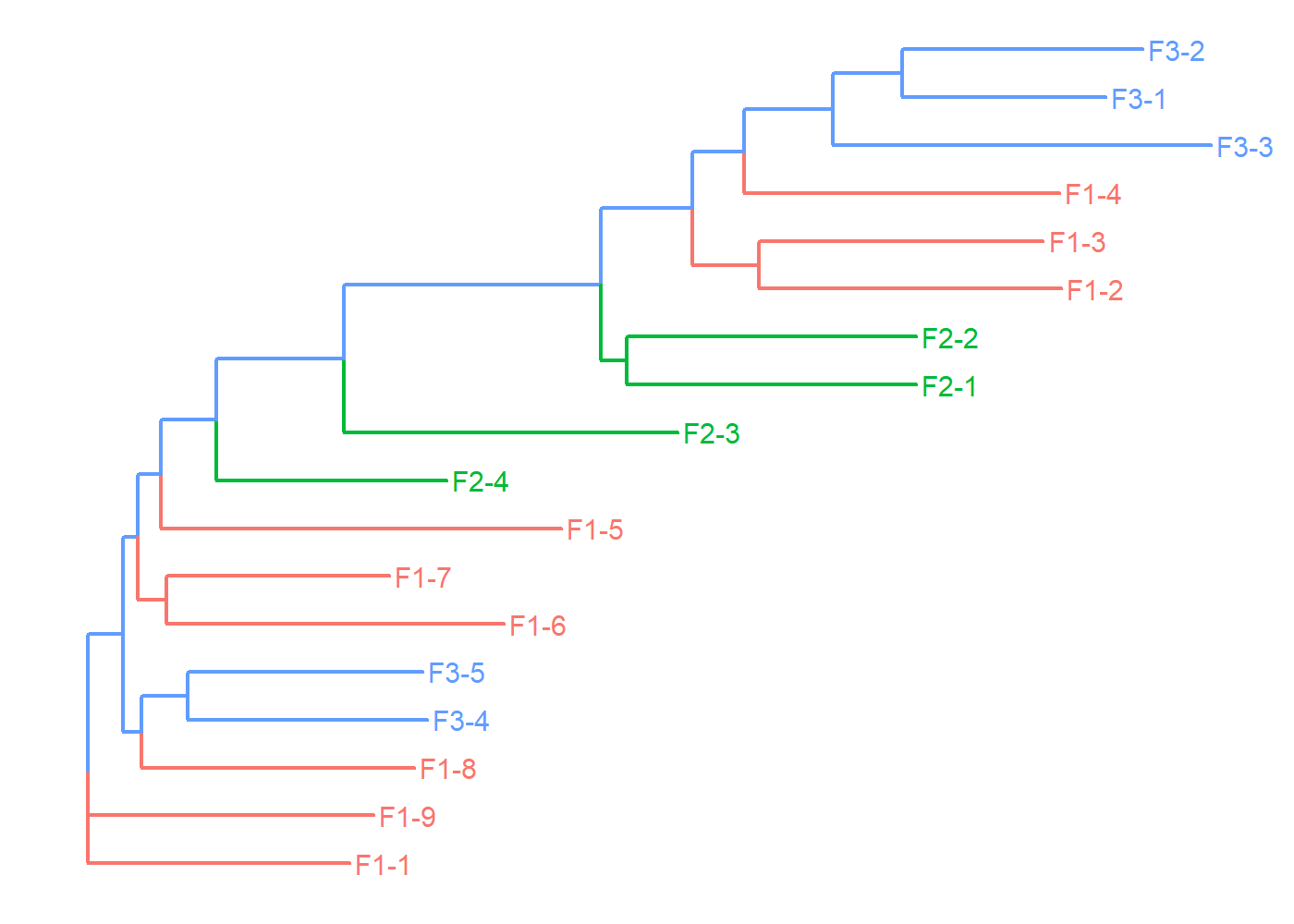


### Figure S3 Phylogenetic relationship of 18 *A. chinensis* bugs with three successive inbreeding generations.


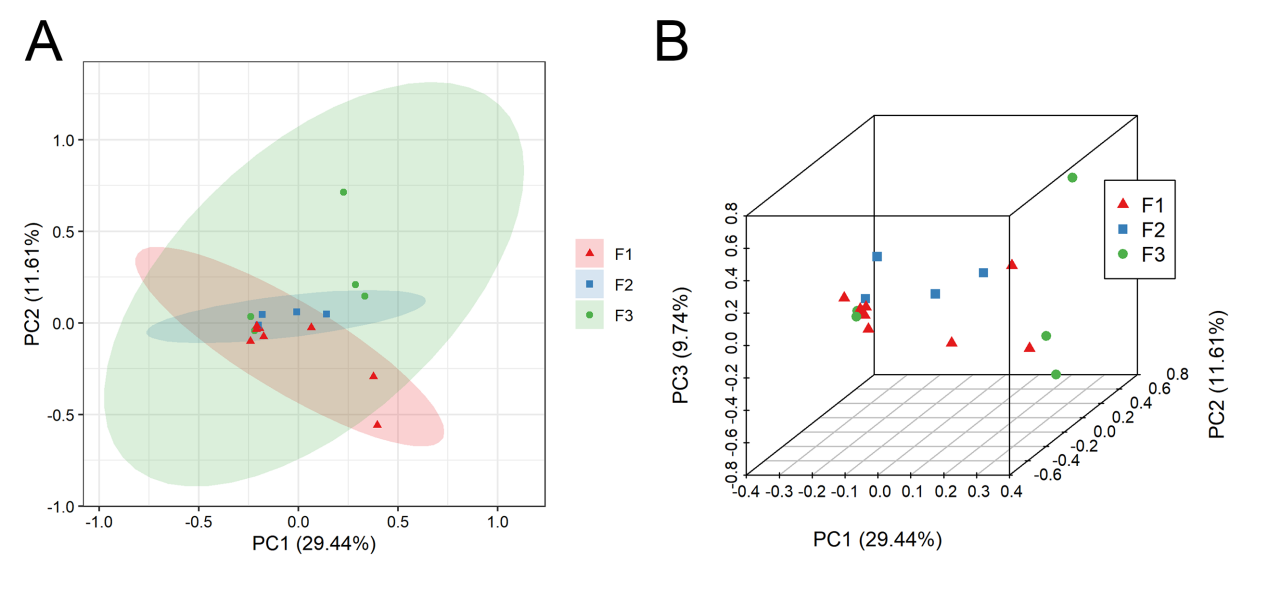


### Figure S4 PCA analyses of 18 *A. chinensis* bugs with three successive inbreeding generations. (A) The 2-dimensional plot of PCA result. (B) The 3-dimensional plot of PCA result.


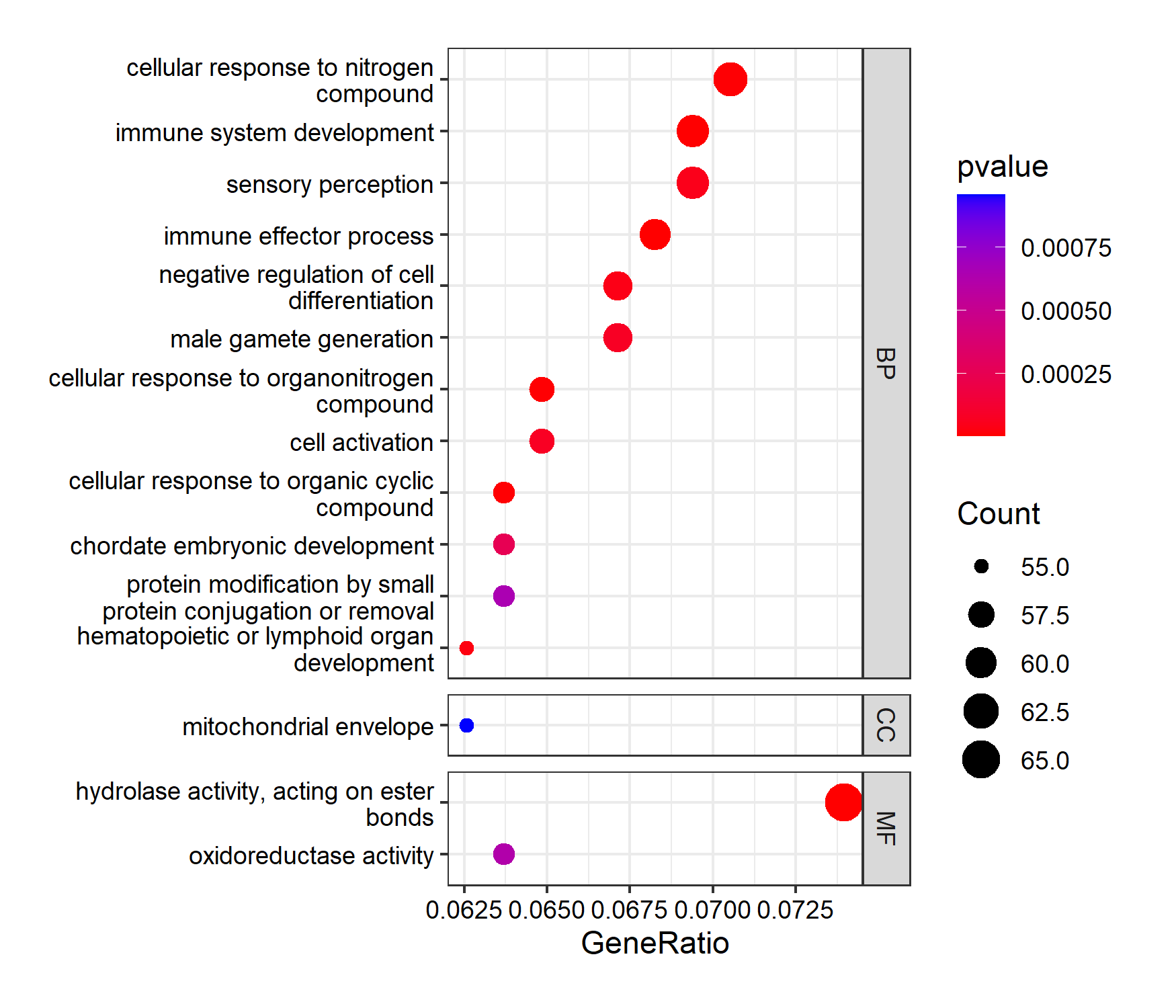


### Figure S5 GO enrichment of genes covered by ROH regions exceeding 400 kb specific to F3 of *A. chinensis*.


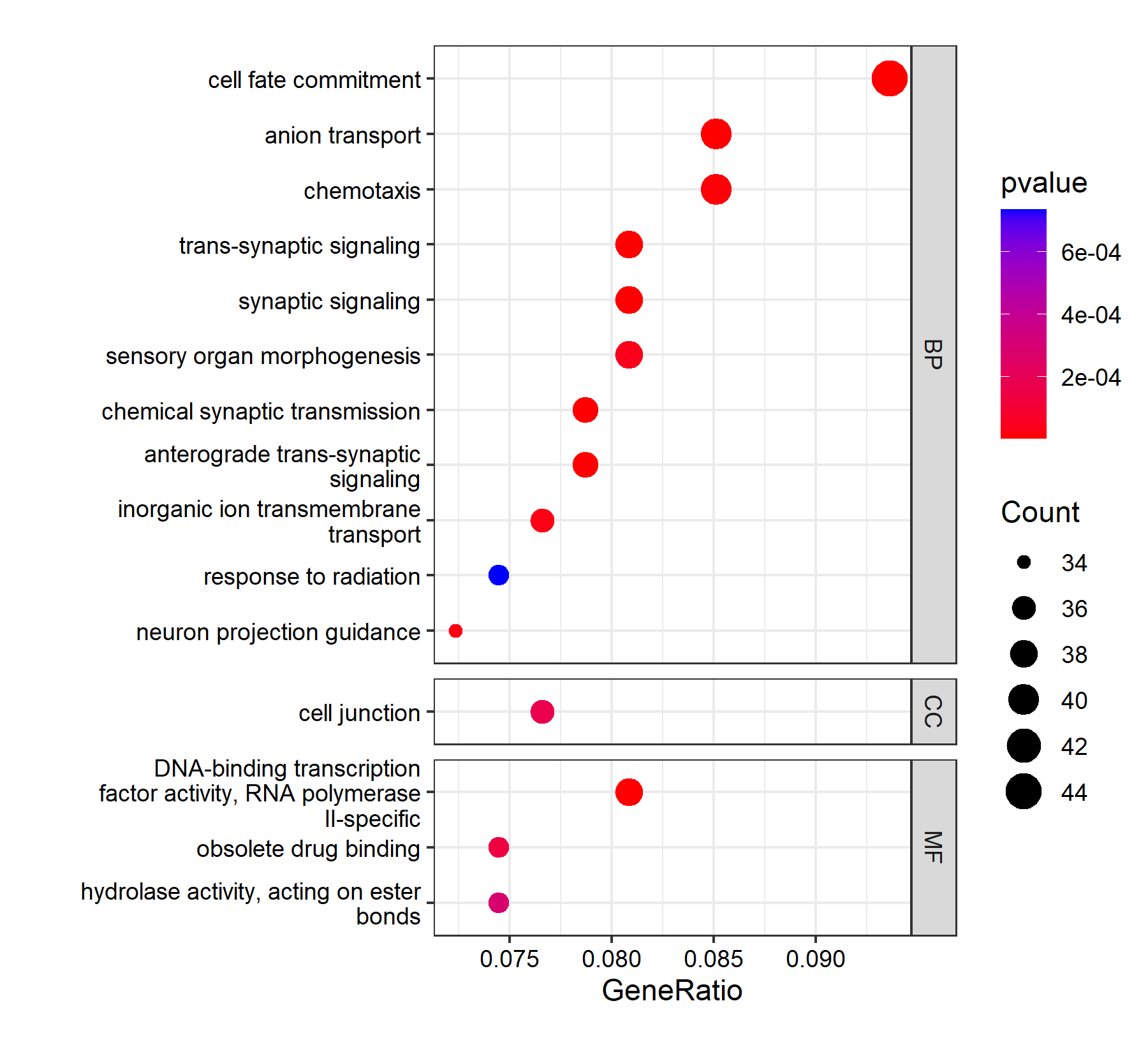


### Figure S6 GO enrichment of genes covered by highly conserved sites (π = 0) specific to F2 and F3 populations of *A. chinensis*.


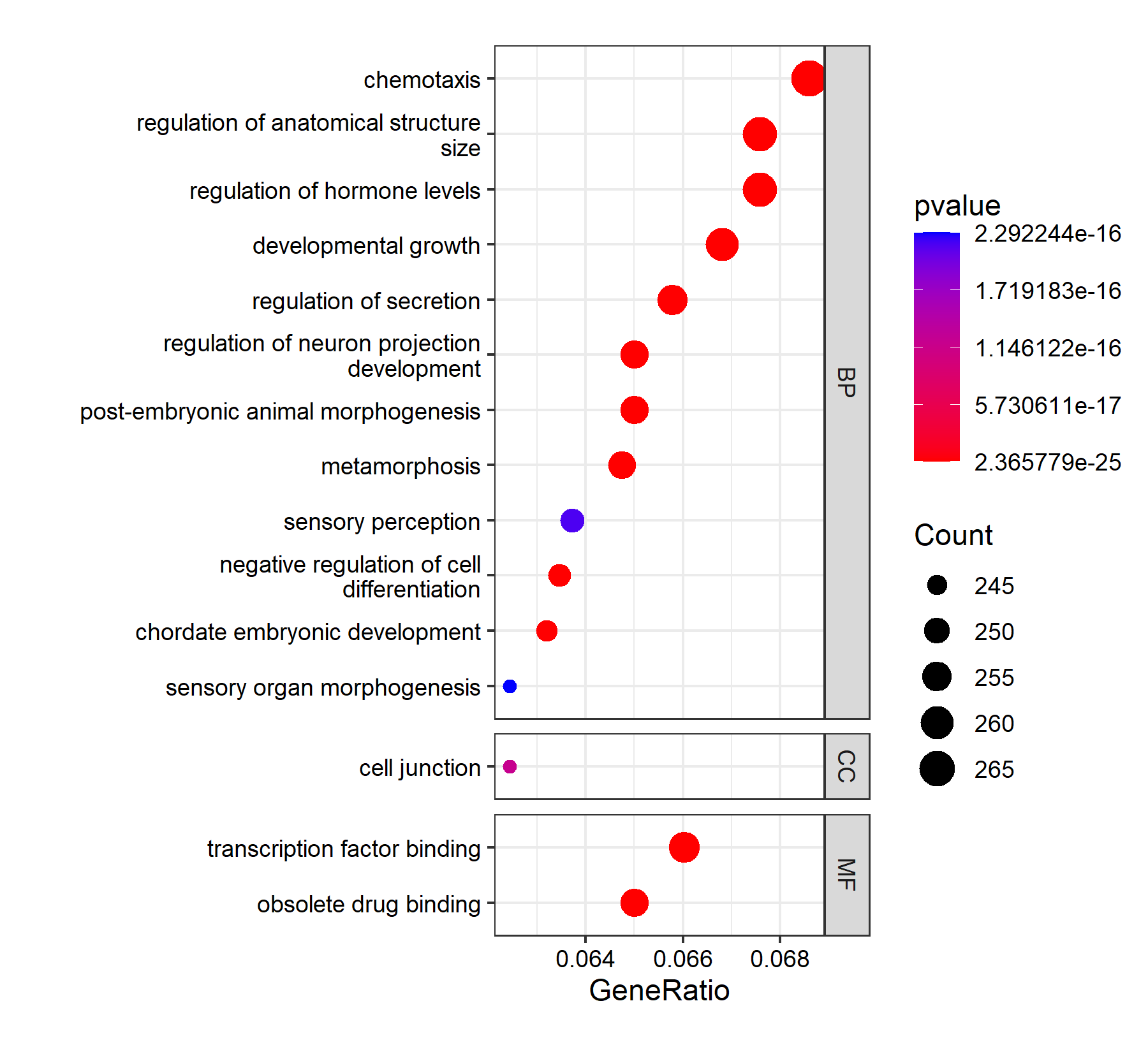


### Figure S7 GO enrichment of genes covered by highly diverse sites (π = 1) specific to F2 and F3 populations of *A. chinensis*.
